# Mechanical Alloying Integrated with Cold Spray Coating for Fabrication Cu_50_(Ti_50−x_Ni_x_), x; 10, 20, 30, and 40 at.% Antibiofilm Metallic Glass Coated/SUS304 Sheets

**DOI:** 10.3390/nano12101681

**Published:** 2022-05-14

**Authors:** Ahmad Aldhameer, Mohamed Sherif El-Eskandarany, Mohamed Kishk, Fahad Alajmi, Mohmmad Banyan

**Affiliations:** 1 Biotechnology Program, Environment & Life Science Research Center, Kuwait Institute for Scientific Research, Safat 13109, Kuwait; mwaheed@kisr.edu.kw; 2 Nanotechnology and Advanced Materials Program, Energy and Building Research Center, Kuwait Institute for Scientific Research, Safat 13109, Kuwait; msherif@kisr.edu.kw (M.S.E.-E.); ftajmi@kisr.edu.kw (F.A.); mbanyan@kisr.edu.kw (M.B.)

**Keywords:** metallic glasses, high-energy ball milling, solid-state coating, thermal stability, nanoindentation, antibacterial

## Abstract

Antibacterial agents derived from conventional organic compounds have traditionally been employed as a biofilm protective coating for many years. These agents, on the other hand, often include toxic components that are potentially hazardous to humans. Multiple approaches have been investigated over the last two decades, including the use of various metallic and oxide materials, in order to produce a diverse variety of usable coating layers. When it comes to material coating approaches, the cold spray technique, which is a solid-state method that works well with nanopowders, has shown superior performance. Its capacity to produce unique material coating in ways that are not possible with other thermal methods is the primary reason for its importance in contemporary production. The present work has been addressed in part to explore the possibility of employing mechanically alloyed Cu_50_(Ti_50−x_Ni_x_)_x_; x = 10, 20, 30, and 40 at.% metallic glass powders, for producing an antibiofilm/SUS304 surface protective coating, using the cold spray approach. In this study, elemental Cu, Ti, and Ni powders were low-energy ball milled for 100 h to fabricate metallic glassy powders with different Ni contents. The as-prepared metallic glassy powders were utilized to coat SUS304 sheets, using the cold spraying process. With high nanohardness values, the as-fabricated coating material, in particular Cu_50_Ti_20_Ni_30,_ demonstrated remarkable performance in comparison to other materials in its class. Furthermore, it displayed excellent wear resistance while maintaining a low coefficient of friction, with values ranging from 0.32 to 0.45 in the tested range. E. coli biofilms were formed on 20 mm^2^ SUS304 sheet coated coupons, which had been injected with 1.5 108 CFU mL^−1^ of the bacterium. With the use of nanocrystalline Cu-based powders, it is feasible to achieve considerable biofilm inhibition, which is a practical strategy for accomplishing the suppression of biofilm formation.

## 1. Introduction

Due to their outstanding processibility, mechanical properties, corrosion resistance, and biocompatibilities, titanium (Ti) metal and its alloys [1] are extensively employed in the orthopedic and prosthodontic fields, among the other known engineering and structural applications [2,3,4]. Unfortunately, as a bioinert material, elemental Ti and its alloys do not have bactericidal potential. In spite of their attractive and pioneering properties, as a bioinert material, Ti and Ti-based alloys do not have bactericidal competence; yet, dental plaque may be found surrounding the implanted dentures quickly after the implantation since they are bioinert materials, not biocidal materials. It has been pointed out that, despite the fact that titanium is a bioinert material, dental plaque may be found around implanted dentures very soon after the implantation [5,6] because they are a kind of bioinert material [7]. In addition to being bioinert and devoid of antibacterial properties, pure Ti does not form a strong connection with soft or hard tissue, which has an impact on the use of such materials in biomedical applications [8].

As a rule of thumb, when bacteria sticks to the surface of biomaterials, the implant-related infection spreads rapidly, resulting in a significant clinical concern [9]. Infections often begin with bacterial adhesion to the implant surface of the biomaterial, which is followed by colonization of bacteria on the implant surface that eventually results in the formation of a biofilm as the colonization grows in size [10,11,12].

### 1.1. Surface Engineering

Surface engineering is concerned with the design and modification of bulk materials’ surfaces (substrates) in order to impart physical, chemical, and technical characteristics to the bulk materials that were not inherent in the bulk materials in the first place [13]. This surface modification step has the potential to improve a wide range of properties, including wear, oxidation, and corrosion resistance, friction coefficients, bio-inertness, electrical characteristics, and thermal insulation, to name a few examples [14]. In the oil and water industries, as well as the medical and food industries, a surface protective coating is the critical application of powder technology because it protects structural materials from corrosion and erosion. Surface protective coating is also used in the pharmaceutical and food industries [15].

### 1.2. Surface Protective Coating

Improvements in surface qualities may be produced by the use of metallurgical, mechanical, or chemical techniques. As a well-known process, coating is simply defined as a deposit of single or multilayered materials artificially coated on the surface of a bulk object (substrate) made of another material. Coating is used to obtain some required technical or decorative properties in part, as well as to protect the material from the expected chemical and physical interactions with its surrounding environment [16]. There are different approaches and technologies that can be employed to deposit the appropriate surface protective layer with different thickness, ranging from a few micrometers (below 10 to 20 µm) to more than 30 µm and up to several millimeters. In general, the coating process can be classified based on the following two categories; (i) the wet-coating approach; such as electroplating, electroless plating and hot-dip galvanizing methods, and (ii) dry-coating approach; such as brazing, weld overlays, physical vapor deposition (PVD), chemical vapor deposition (CVD), thermal spray techniques and, most recently, the cold spray technique [4].

#### 1.2.1. Antibiofilm Surface Protective Coating

It has been agreed that as soon as a biofilm has developed, it becomes extraordinarily resistant to antibiotics and the immune system of the donor, which makes it difficult to remove the biofilm from the implant surface, resulting in further complications. To prevent both the spread of microorganisms and material deterioration, antimicrobial surfaces that inhibit biofilm growth are the most effective method for preventing biofilm development. In recent decades, a range of strategies have been developed in order to acquire this antimicrobial characteristic, which may be grouped into three major categories. The first is the creation of an anti-adhesive surface that, by physical or chemical modification, prevents bacteria from adhering to it and forming biofilms as a result of the adhesion [17]. The second strategy involves building coatings that allow antimicrobial chemicals to be delivered in highly concentrated and customized quantities exactly where they are required. This is accomplished by developing coatings that are resistant to bacteria [18]. As a result, toxicity and resistance development are less likely to occur [18]. Third, the use of biocidal (or contact-killing) coatings, in which bactericidal chemicals are fixed on the surface to provide long-term protection against bacterial contamination [19]. All three strategies are capable of imparting an antimicrobial effect on the coated surface; however, they each have their own set of drawbacks that should be taken into consideration when developing a strategy for application [19].

The usage of implants that have antibacterial activity has been investigated from the perspective of implant materials in order to reduce the risk of infection in the patient. Implants with antibacterial surface coatings such as Ti6Al4V, MoS_2_,and TiO_2_ [20,21,22] are an excellent example of this kind of technology. Throughout the course of many decades of study, surface modification has been proved to be an effective means of producing implants with antibacterial activity. With the exception of Zn [23], antibacterial agents such as Ag [24,25,26], and Cu [27] were often used in this respect.

#### 1.2.2. Cold Spray Coating

**Figure 1:**
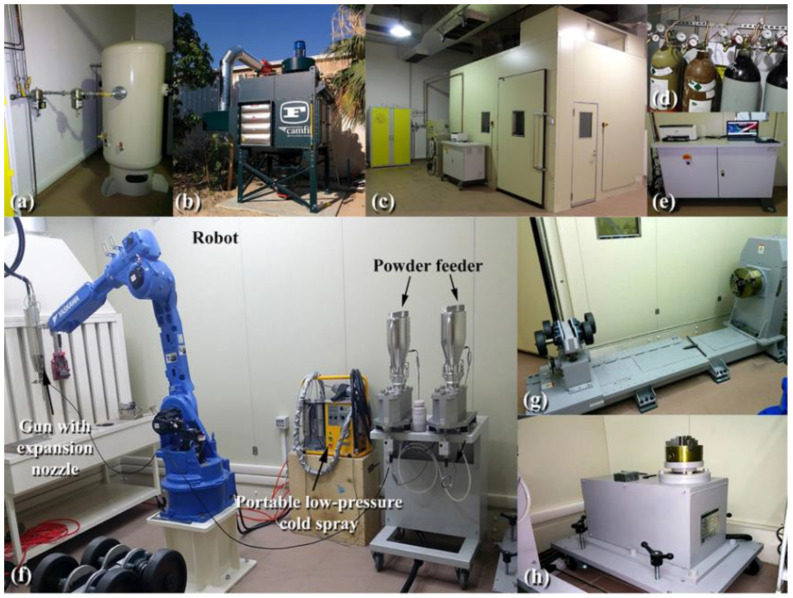
Fully automated cold spray system housed in Nanotechnology Laboratory, KISR used for coating of stainless-steel (SUS 304) sheets with metallic glassy Cu_50_(Ti_50−x_Ni_x_) alloy powders. The cold spray components are (**a**) pressurized air reservoir, (**b**) filtration system, (**c**) completely sealed cold spray room, (**d**) argon/nitrogen/compressed air gas cylinders, and (**e**) control unit, (**f**) main components including powder feeders, a robot, low-pressure cold spray system for outdoor coating, (**g**) horizontal turntable, and(**h**) vertical turntable (for coating the outer and inner parts of the objects).

**Figure 2:**
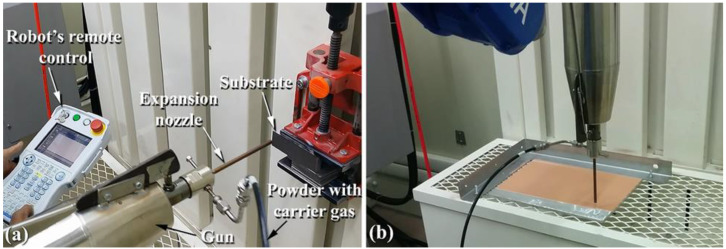
The gun with the expansion nozzle of a cold spraying system can be oriented using a robot system in (**a**) horizontal or (**b**) vertical positions.

In the mid-1980s, a newcomer to the thermal spray family was introduced: the cold gas-dynamic spray process (also known as cold spray), which was developed at the Institute of Theoretical and Applied Mechanics of the Russian Academy of Sciences in Novosibirsk [28] and introduced into the thermal spray family. Cold gas spray, micro cold spray, cold gas dynamic spray, kinetic spray, and supersonic particle deposition are some of the various names that are used for the same procedure. In contrast to the typical thermal spray approach, the cold spray technique is a solid-state process in which the powder feedstock does not melt and stays in its solid state throughout the operation. Figure 1 and Figure 2 display digital photos of a typical cold spray system used in the present work.

### 1.3. Aim of the Present Study

The majority of the instruments used in the medical and food industries are made of austenitic stainless steel alloys (SUS316 and SUS304), which possess a high chromium content ranging between 12 and 20%. It is widely recognized that the use of chromium metal as an alloying element in steel alloys significantly improves the corrosion resistance of such a traditional alloy. In contrast to their great corrosion resistance, stainless steel alloys SUS316 and SUS304 do not have significant antibacterial properties [29,30]. Consequently, infection and inflammation, which are often caused by the adhesion and colonization of bacteria on the surfaces of stainless steel biomaterials, might be anticipated. Having a substantial issue associated with the bacterial adhesion and biofilm formation pathways may result in considerable problems, which can lead to a decline in health with a variety of effects that can directly or indirectly influence human health.

The current study is part of a project (EA074C) funded by the Kuwait Foundation for the Advancement of Sciences (KFAS), under Contract number: 2010-550401 to investigate the possibility of employing a mechanical alloying (MA) technique for the fabrication of high-quality powders of metallic glassy Cu_50_(Ti_50−x_Ni_x_) alloy powders (x = 10, 20, 30, and 40 at.%), for producing an antibiofilm/SUS304 surface protective coating, using the cold spray approach. The effect of Ni content on the mechanical properties such as nanohardness, Young’s modulus and the tribology of the coated SUS304 sheets are presented. Additionally, the antibacterial behavior of the metallic glassy material coating on stainless steel substrates, is discussed. In the second part of this project, the detailed electrochemical corrosion properties will be examined and published.

## 2. Materials and Methods

### 2.1. Preparations of Cu-Based Metallic Glassy Alloy Powders by Mechanical Alloying

**Figure 3:**
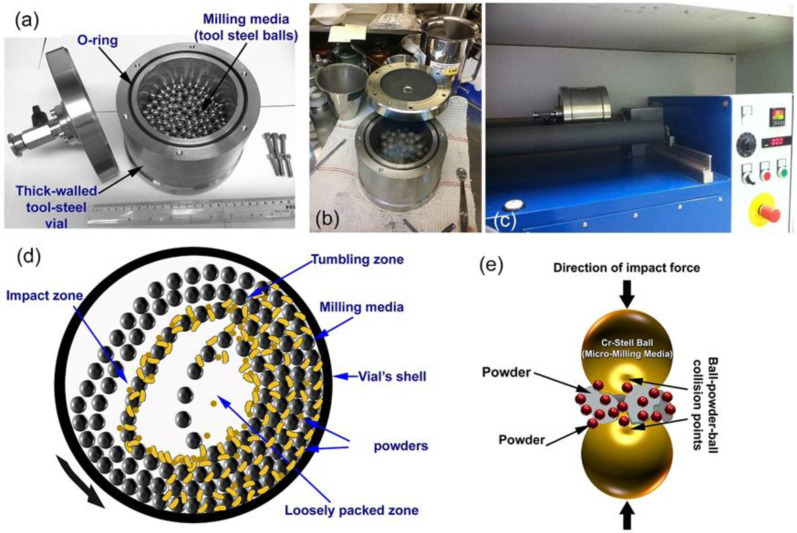
Photos taken from the Nanotechnology Laboratory in Kuwait Institute for Scientific Research (KISR) display set-up of a lab-scale roller mill (1000 m in volume) that was used for preparation of metallic glassy Cu_50_(Ti_50−x_Ni_x_) alloy powders (x; 10, 20, 30, and 40 at.%). (**a**) Tool-steel milling tool consists of 1000 mL cylindrical vial and 100 tool-steel balls (12 mm in diameter), (**b**) the precursor elemental Cu, Ti, and Ni were hand mixed in a glove box under He atmosphere and charged into the vial, and (**c**) the vial was mounted on a roller mill, where the ball milling process was undertaken at 250 rpm. The configuration and movement of the balls within the vial of tumbling mill in the dynamic mode is shown schematically in (**d**), whereas a typical ball-powder-ball collusion for a low energy tumbling ball mill is illustrated in (**e**).

For the purpose of the present work, pure metallic alloying elements of Cu, Ti, and Ni powders (above 99.9 wt.%, less than 20 µm in diameter) were used as starting reactant materials. The powders were firstly balanced to give the average nominal composition of the starting charge of Cu_50_(Ti_50−x_Ni_x_), where x = 10, 20, 30, and 40 at.%, and mixed, using an agate mortar and pestle inside a glove box (UNILAB Pro Glove Box Workstation, mBRAUN, Germany) filled with helium gas. An amount of 150 g of the powders were then charged into a Cr-steel vial (~1000 mL in volume, Figure 3a) and sealed together with 100 balls (14 mm in diameter) made of Cr-steel alloy in the glove box, as displayed in Figure 3b. The ball-to-powder weight ratio was selected to be 36:1. The MA process was started by mounting the vial on a roller mill (RM20) provided by Zoz GmbH, Germany, operated at room temperature with a rotation speed of 235 rpm (Figure 3c). The progress of the solid-state reaction was monitored by interrupting the MA process after selected ball milling time (12.5, 25, 50, 75, and 100 h), where the vial was opened in the glove box to take a represented sample. All samples were then characterized by different analytical techniques.

During ball milling of the metallic powders, the useful kinetic energy that can be applied to the powder particles was governed by the following factors: (i) collision between the balls and the powders (Figure 3d,e), (ii) pressure loading of powders pinned between milling media or between the milling media and the liner (Figure 3d), (iii) impact of the falling milling media (Figure 3d), (iv) shear and abrasion caused by dragging of particles between moving milling media (Figure 3e), and shock wave transmitted through crop load by falling milling media (Figure 3d).

### 2.2. Fabrication of Cu-Based Metallic Glassy Powder Coated/SUS304 Composites by Cold Spray Process

**Figure 4:**
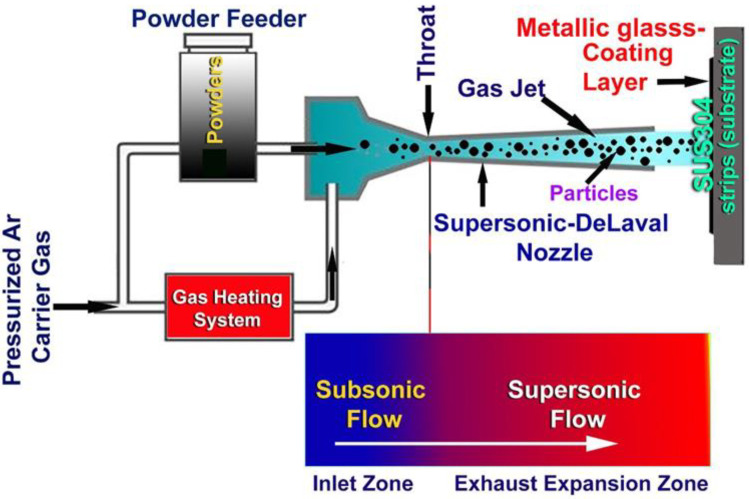
Schematic of cold spray process for coating SUS304 sheet with Cu_50_(Ti_50−x_Ni_x_) (x = 10, 20, 30, and 40 at.%) metallic glassy powders.

Due to the fact that the as-prepared powders were noncrystalline (amorphous) powders, it was predicted that they would crystallize into stable (crystalline) phase when heated beyond their crystallization temperature. Because the purpose of this work is to investigate the influence of Cu-based metallic glassy alloy powders on the development of biofilms, the glassy phase must be maintained throughout the spraying process. As a consequence, SUS304 sheets were coated on both sides using a cold spraying process. Stainless steel (SUS304) sheets, which were firstly rinsed with acetone and ethanol, dried in an oven at 150 °C for 1 h were used as substrate metal. The substrate’s surface was treated by alumina blasting at room temperature before the coating process. Unlike the thermal spray combustion-based methods, the cold spray approach is achieved at low temperature (in the range between 100 to 900 °C), being far below the melting points of the feedstock powders. In the present work, the cold spraying process was started at low temperature (400 °C) with a supersonic jet processed at a very high velocity (1200 m/s). The powders were charged in cold spray feeder (Figure 1f) and subjected to high pressure argon gas flow to pass through pipeline connected to a supersonic jet and then sprayed onto the surface of stainless steel substrate, as presented in Figure 4. This process was repeated 5 times on each face of the sheet.

### 2.3. Material Characterizations

#### 2.3.1. Structural Characterizations

The general structural changes upon ball milling of Cu-based master alloys were investigated with X-ray diffraction (XRD, Rigaku, Kawasaki, Japan), using Rigaku-SmartLab 9 kW equipment. All the samples were analyzed with a speed of 2 degree/min via continuous 2θ/θ scan mode, using CuKα radiation (λ= 0.15418 nm) operating at 45 kV 200 mA. A high-speed 1D X-ray detector D/teX Ultra 1D mode (D/teX) with Ni Filter was used. The diffraction patterns were obtained over the 2θ range of 20° to 80°, with a step size of 0.02/2θ and a time of 1 s/step. The XRD resulted from constructive and destructive interference caused by scattering of X-rays from atoms in a regular array, with diffraction lines appearing at angles that satisfy Bragg’s approach.

Field emission high-resolution transmission electron microscopy (FE-HRTEM) equipped with energy-dispersive X-ray spectroscopy (EDS) of as-synthesized materials powder samples were carried out using 0.17-nm resolution JEOL microscopes of JEOL 2000F operated at 200 kV.

The sample powders were dissolved into ethanol, and then, a few drops of the suspension were dropped onto a copper (Cu)-microgrid and dried in a dictator. The microgrid was then mounted into the TEM transfer rod and placed in the vacuum sample chamber of TEM. The micrographs for the bright field image (BFI), dark field image (DFI), and selected area electron diffraction patterns (SADPs) were collected, where EDS was used for elemental analysis.

#### 2.3.2. Morphological Characterizations and Elemental Analysis

Field emission scanning electron microscopy (FESEM/EDS), using JEOL: JSM-7800F (JEOL, Musashino, Akishima, Tokyo 196-8558, Japan), operated at an acceleration power of 15 kV, was used to investigate the morphological characterizations of the samples and their elemental compositions. The powder samples were placed on double-sided adhesive carbon tape and placed on a Cu-sample holder. The samples prevented any possible charging in the image and kept the powder steady. The samples were inserted into the FE-SEM chamber for analysis. The concentrations of the metallic alloying elements in the as-ball milled powders were determined by both of TEM/EDS, and SEM/EDS techniques.

#### 2.3.3. Nanoindentation

This technique was employed to determine the nanohardness and Young’s modulus of as-coated samples, using a Buruker Nanoindenter (Los Angeles, CA, USA) with diamond Berkovich-tip. This advanced approach allowed the investigation of the degree of uniformity in the coated layers at the sub-microscale. The test was achieved by the single indent continuous multicycle ramp approach with an applied force of 400 mN.

#### 2.3.4. Thermal Stabilities

Shimadzu Thermal Analysis System /TA-60WS (Shimadzu, Nishinokyo Kuwabara-cho, Nakagyo-ku, Kyoto 604-8511, Japan), using a differential scanning calorimeter (DSC) was employed to investigate the thermal stability of the as-ball milled powders, indexed by the transition glass, crystallization (T_x_), and enthalpy change of crystallization (ΔH_x_), using a heating rate of 40 °C/min.

#### 2.3.5. The Tribology

The tribological behavior of the metallic glassy coatings was characterized by a pin-on disc tribometer (MICROTEST, S.A, Madrid, Spain) against a hard ball (10 mm in diameter) made of WC-Ni composite. The sliding speed of the specimen relative to the ball was 5 × 10^−2^ m/s, the sliding distance was 100 m with a total sliding time of 950 s, and the normal load applied load was 10 N. The coefficient of friction (CPF) of the sliding couple was continuously recorded during the test. The surface of the samples was examined after the wear test, using the SEM/EDS approach.

### 2.4. Bacterial Strain

#### 2.4.1. Biofilm Growth Conditions

*Escherichia coli* (ATCC 25922) was used as a test organism. Biofilms were grown, with the following modification; planktonic *E. coli* cells were grown in brain heart infusion (BHI) (Oxoid, UK). Biofilms were grown on 22 mm^2^ coated SUS304 coupons. Sterile coupons were positioned vertically in 50 mL conical tubes (BD Falcon, Franklin Lakes, NJ, USA) with 6 mL prewarmed BHI. Tested coupons were inoculated with 100 μL 0.5 McFarland standard suspensions (equivalent to 1.5 × 10^8^ CFU mL^−1^) of a 24 h culture. Biofilms were left to grow for various periods of time (24, 48 and 72 h) at 37 °C.

#### 2.4.2. Effect of Cu-Ti-Ni-Coating on Biofilm Formation

The biofilm growth assays following the earlier published data by our research group, briefly, 24, 48 and 72h old biofilm were used as previously described. Triplicate coupons were taken to prepare biofilms, 22 mm^2^ Cu_50_Ti_20_Ni_10_ coating/SUS304 coupons were rinsed in phosphate buffer solution (PBS) to remove non adherent cells and placed in BHI (7 mL). The tested coupons were vortexed for 15 min to remove the biofilm cells. Viable bacteria were then enumerated.

## 3. Results and Discussions

### 3.1. Crystal Structure

**Figure 5:**
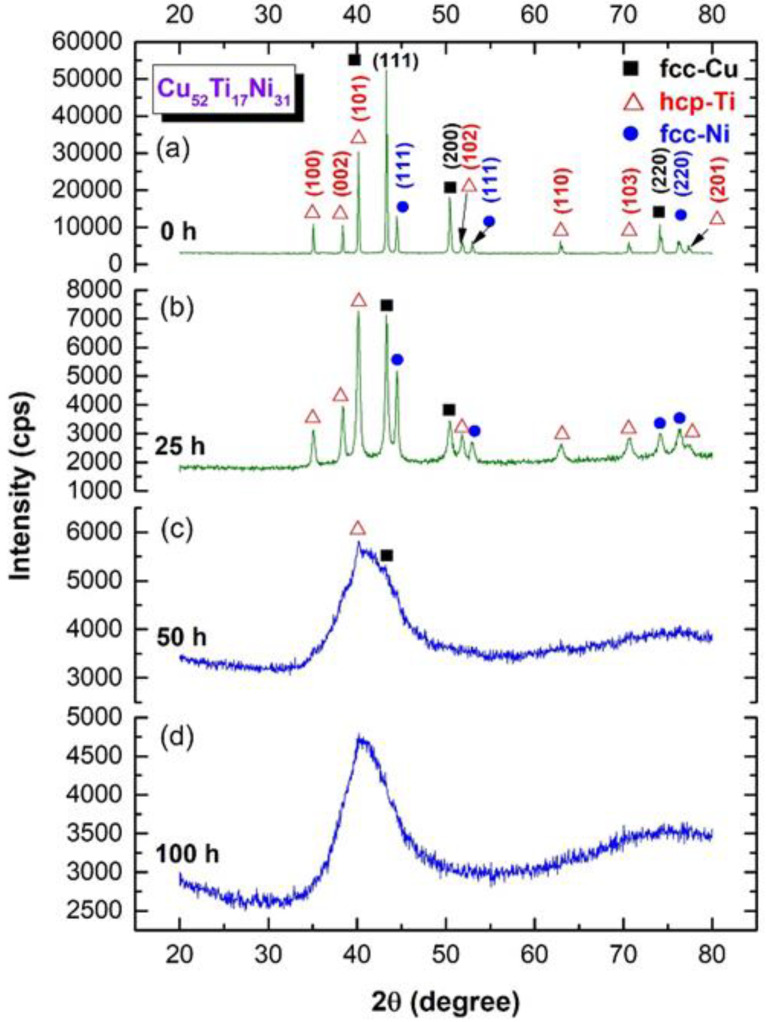
X-ray diffraction (XRD) patterns of ball milled Cu_52_Ti_17_Ni_31_ powders obtained after ball milling for (**a**) 0 h, (**b**) 25 h, (**c**) 50, and (**d**) 100 h.

The X-ray technique was used to monitor the progress of the solid-state reaction between elemental Cu, Ti, and Ni powders and was undertaken after several stages of MA time. Figure 5 displays the XRD patterns of Cu_52_Ti_17_Ni_31_ powders obtained after different amounts of MA time.

**Figure 6:**
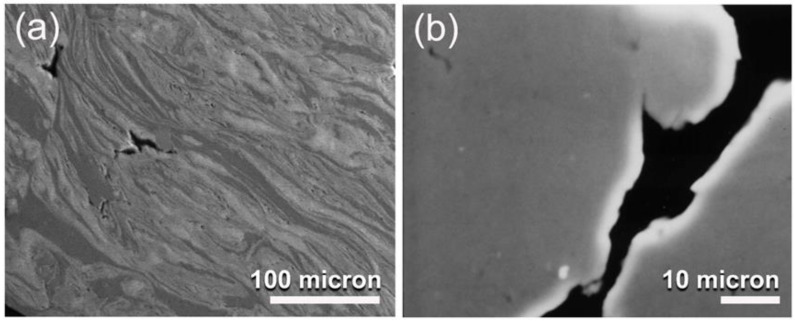
Micrographs of field-emission scanning electron microscope (FE-SEM) for mechanically alloyed Cu_52_Ti_17_Ni_31_ powders obtained after (**a**) 25 h, and (**b**) 100 h of ball milling time.

At the starting stage of MA (0 h), the powders were composed of large polycrystalline grains of the alloying elements, indicated by the sharp Bragg peaks of fcc-Cu (PDF# 00-004-0836), hcp-Ti (PDF# 00-005-0682), and fcc-Ni (PDF# 00-004-0850), as shown in Figure 5a. Increasing the MA time (25 h) led to enhance the mechanically-induced grain refining of the powders, as indicated by the significant broadening of the Bragg lines with a dramatic decrease in their intensities (Figure 5b). During this stage of milling, the powder particles were subjected to continuous shear forces generated by the ball milling tools (Figure 3d,e), leading to the formation of multi-intimated layers of the elemental powders, as displayed in Figure 6a. 

**Figure 7:**
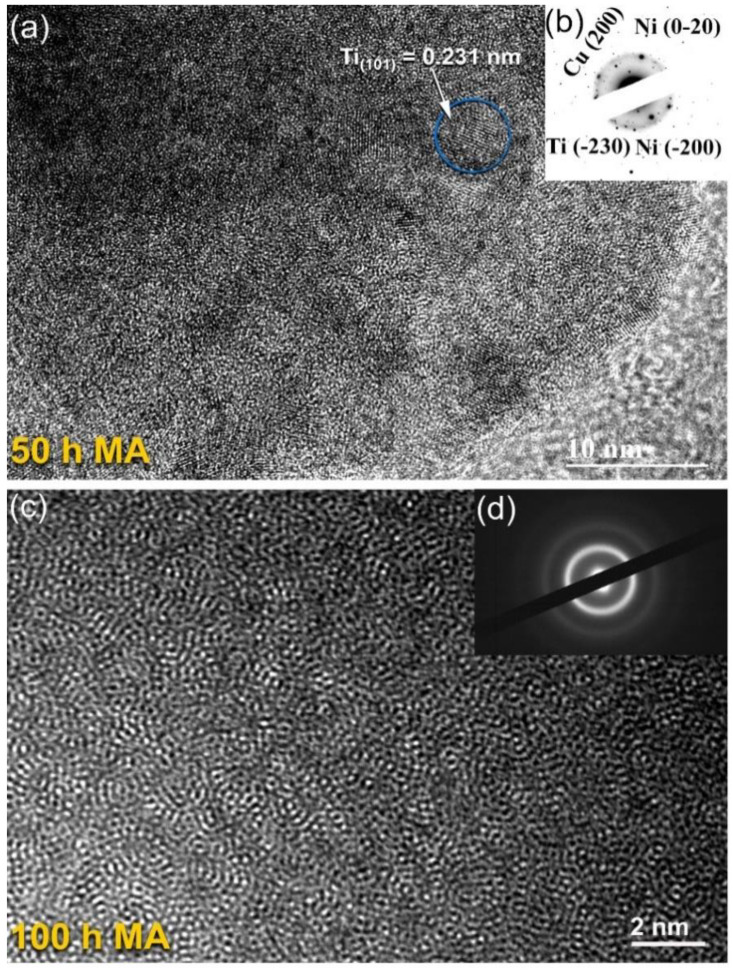
Field-emission high resolution electron microscope (FE-HRTEM) images of Cu_52_Ti_17_Ni_31_ powders obtained after (**a**) 50 h, and (**c**) 100 h of ball milling time. The corresponding selected area diffraction pattern (SADP), and nano beam diffraction pattern (NBDP) for (**a**), and (**c**) are displayed in (**b**), and (**d**), respectively.

The addition of further MA time (50 h) resulted in an increase in the mechanically-induced solid-state reactions of the alloying elements, as seen by the disappearance of the minor Bragg peaks corresponding with the pure elemental powders, as shown in Figure 5c. Furthermore, a significant reduction in the intensities related with the principal Bragg peaks of Cu(111), Ti(001), and Ni(111) were accomplished. As shown in Figure 5c, clear first (35° to 48°) and second (64° to 80°) broad diffuse haloes were observed, indicating the formation of an amorphous phase that coexisted with unprocessed nanocrystalline Cu and Ti powders. The corresponding HRTEM image of this sample revealed a mazing-like morphology (Figure 7a), implying the formation of an amorphous phase coexisting with unprocessed nanocrystalline powders, as indexed in Figure 7a,b.

According to Figure 5d, the XRD pattern of the powders produced at the end-stage of ball milling (100 h MA) exhibited a uniform diffuse halo pattern. Furthermore, the Bragg peaks associated with the initial feed stock crystalline powders were no longer present, indicating that the mechanically induced solid-state reaction had been completed and that a single amorphous phase had been formed. In parallel, the layer-like morphology had disappeared and the sample obtained after 100 h of MA had a mirror-like morphology, as shown in Figure 6b. This indicates the absence of a multiphase structure and the formation of a uniform product.

HRTEM images along with the matching NBDP measurements of the powders obtained after 100 h of MA are presented in Figure 7c,d, respectively. According to Figure 7c, the sample seemed featureless and uniform in its internal structure, with no evidence of any crystalline phases precipitating from the sample’s surface or inside it. Furthermore, the powders displayed a maze contrast of a densely packed metallic amorphous phase, indicating the fabrication of an amorphous phase at the nanoscale level. The NBDP (Figure 7d), again exhibited a characteristic halo diffraction of an amorphous phase, with no sharp rings and/or spots associated with the unprocessed crystalline powders.

**Figure 8:**
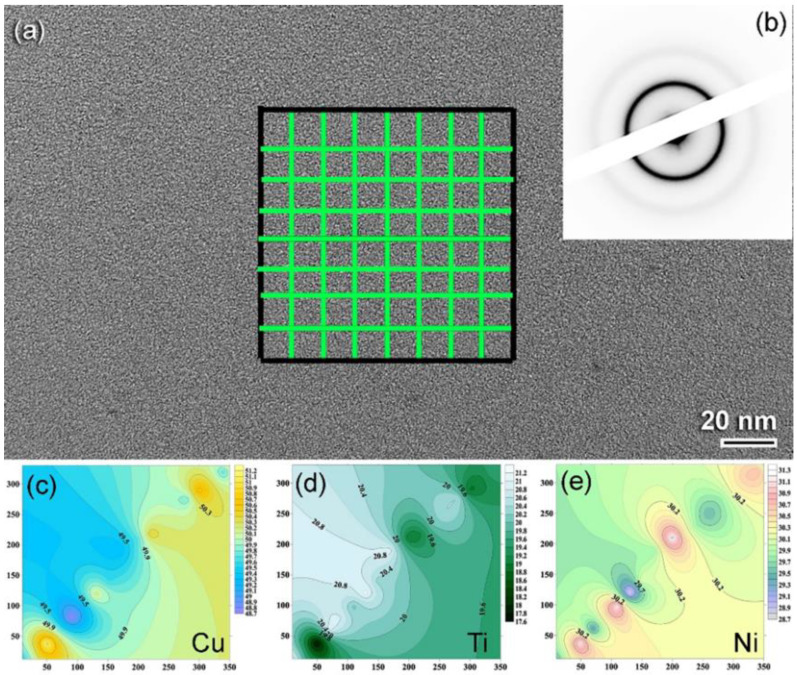
(**a**) Image of field-emission high resolution electron microscope (FE-HRTEM), and (**b**) nanobeam diffraction pattern (NBDP) of metallic glassy Cu_50_Ti_20_Ni_30_ powders obtained after 100 h of milling time. The area indexed in (**a**) refers to those regions that were examined by energy dispersive X-ray spectroscopy (EDS) to investigate the local composition of the prepared powders. The isochemical contour maps corresponding to Cu, Ti, and Ni metals are displayed in (**c**–**e**), respectively.

HRTEM integrated with energy dispersive X-ray spectroscopy (EDS) was used to realize the chemical composition and homogeneity of as-fabricated Cu_50_Ti_20_Ni_30_ metallic glass obtained after 100 h of milling. Figure 8a,b show a high-resolution transmission electron microscopy (HRTEM) image and a nanobeam diffraction pattern (NBDP) of the powders following the last step of milling. As shown in Figure 8a, increasing the MA period to 100 h did not result in any phase transformations, and the milled powders maintained their maze-like shape with a leak in crystallinity, as indicated in Figure 8b.

Figure 8a shows an area in the middle section of the figure that has been split into 64 subregions, with EDS analysis being performed at the center point of each subregion, as shown by the indices in Figure 8a. The EDS results were utilized to develop isochemical contour maps for the alloying elements of Cu (Figure 8c), Ti (Figure 8e), and Ni (Figure 8e) based on the results of the experiment. The results revealed that the three alloying elements were distributed very homogeneously beyond the nanoscale and exhibited no apparent separation or aggregation. Moreover, the Cu/Ti/Ni ratio measured from several nanoscaled subregions with EDS analysis showed the average composition of ratio of Cu/Ti/Ni as 52/17/31.

**Figure 9:**
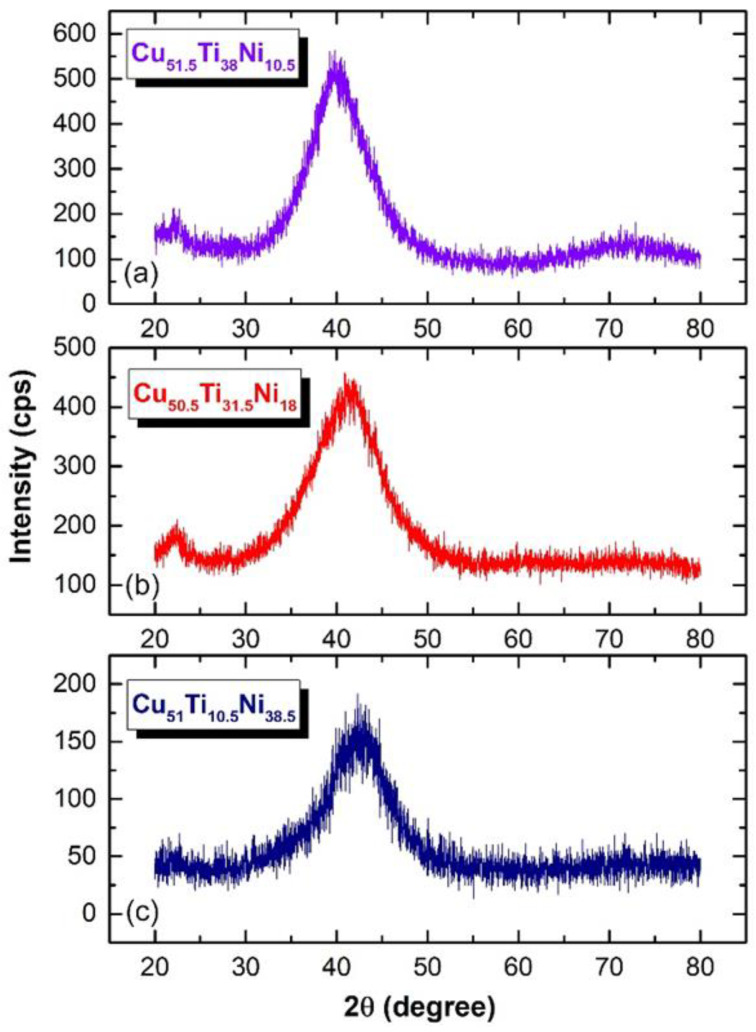
XRD patterns of mechanically alloyed (**a**) Cu_51.5_Ti_38_Ni_10.5_, (**b**) Cu_50.5_Ti_31.5_Ni_18_, and (**c**) Cu_51_Ti_10.5_Ni_38.5_ powders obtained after ball milling of 100 h.

The XRD patterns of mechanically alloyed Cu_51.5_Ti_38_Ni_10.5_, Cu_50.5_Ti_31.5_Ni_18_, and Cu_51_Ti_10.5_Ni_38.5_ powders obtained after 100 h of ball milling are shown in Figure 9a–c, respectively. Likewise, Cu_52_Ti_17_Ni_31_ powders milled for 100 h, all the samples with different compositions revealed a halo-diffuse pattern of amorphous structure.

### 3.2. Thermal Stability

**Figure 10:**
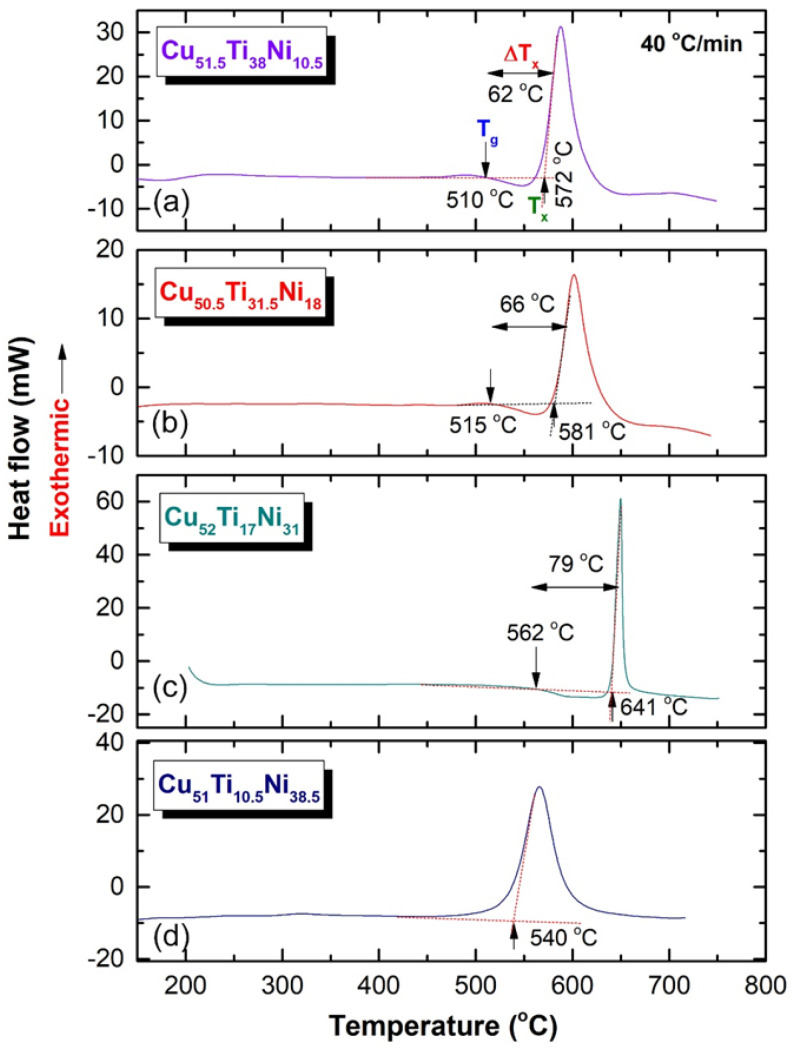
DSC thermograms of (**a**) Cu_51.5_Ti_38_Ni_10.5_, (**b**) Cu_50.5_Ti_31.5_Ni_18_, and (**c**) Cu_52_Ti_17_Ni_31_, and (**d**) Cu_51_Ti_10.5_Ni_38.5_ metallic glassy alloys obtained after ball milling of 100 h.

The thermal stability of the synthesized amorphous powders as a function of Ni content was evaluated using a DSC technique in order to determine the temperature of the cold spray procedure that should be applied when coating SUS304 with the fabricated amorphous powders. The DSC thermograms for all the end-products (100 h) are displayed together in Figure 10. All samples except Cu_51_Ti_10.5_Ni_38.5_ revealed two opposite events in a temperature range lying between 500 to 700 °C, as shown in Figure 10.

**Figure 11:**
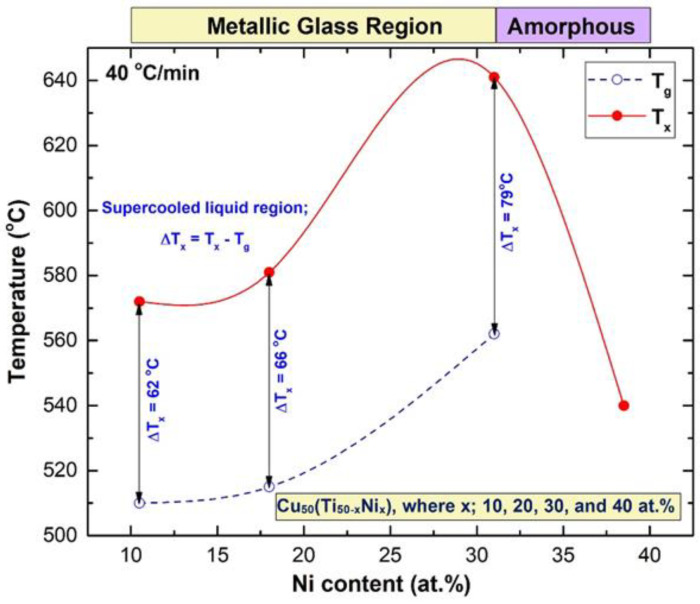
Effect of Ni content (x) on the thermal stability, indexed by T_g_, T_x_, and ΔT_x_ for ternary Cu_50_(Ti_50−x_Ni_x_) (x; 10, 20, 30, and 40 at.%) metallic glassy powders obtained after 100 h of milling.

The first event was an endothermic peak, which appeared for metallic glassy Cu_51.5_Ti_38_Ni_10.5_, (a) Cu_50.5_Ti_31.5_Ni_18_ (b), and Cu_52_Ti_17_Ni_31_(c). As shown in Figure 10, endothermic peaks are formed as a result of the change of amorphous to metallic glass phases at glass transition temperatures (T_g_) in the temperature range of 510 to 562 °C. In the second event, we are referring to those exothermic peaks that formed at crystallization temperature (T_x_) in a temperature range that was substantially higher (572 to 641 °C), as displayed in Figure 10. The supercooled liquid regions (ΔT_x_) are defined as the areas that exist between the two occurrences, i.e., T_g_ and T_x_. The sample Cu_51_Ti_10.5_Ni_38.5_ (Figure 10d) on the other hand, did not exhibit an endothermic event, indicating that the produced phase of this sample was simply an amorphous metastable phase, as shown in Figure 9c. Based on the present results, it can be then concluded that the metallic glass region in Cu_50_(Ti_50−x_Ni_x_) (x = 10, 20, 30, and 40 at.%) was in the range of 10 at.% ≤ × ≥ 31 at.%, as indexed in Figure 11. 

The dependence of thermal stability, characterized by T_g_, T_x_, and ΔT_x_ for Cu-Ti-Ni ternary system on the Ni content (x) was investigated from Figure 10 and plotted in Figure 11. Increasing the values of these parameters indicated the formation of a stable metallic glassy alloy that could resist the applied cold spray temperature below its T_x_. The T_g_, T_x_, and ΔT_x_ monotonically increased with increasing the Ni content (x) in a compositional range of 10 at. % ≤ × ≥ 31 at.%, as shown in Figure 11. 

Based on the results of the present work, the metallic glassy Cu_52_Ti_17_Ni_31_ system exhibited the highest thermal stability, suggested by its highest value of T_g_ (562 °C) and T_x_ (641 °C), and large ΔTx that extended up to 79 °C, as displayed in Figure 10c and Figure 11. The T_g_, T_x_, and ΔT_x_ of Cu_51.5_Ti_38_Ni_10.5_ and Cu_50.5_ Ti_31.5_ Ni_18_ systems revealed lower values of 510, 572, 62 °C, and 515, 581, 66 °C, respectively, as shown in Figure 11. When these values were compared to those found for the Cu_52_Ti_17_Ni_31_ system, it became clear that the latter system was the most suited alloy powder for use in the cold spray coating process, with the former system being the least suitable.

The impact of substituting Ni for Ti on the capacity of the metallic glassy alloy to produce glass resulted in an increase in the supercooled liquid area of the metallic glassy alloy in the range of 10 to 30 Ni at. percent, as seen in Figure 10 and Figure 11. Because this metastable phase is sensitive to crystallization during the cold spray process, it is critical to use metallic glassy alloys with a large supercooled liquid area in order to prevent this phase from crystallization.

Additionally, the addition of Ni additives resulted in an improvement in the thermal stability of the resulting metallic glassy powder, as shown by the high values of the glass transition temperature and the crystallization temperature within the range of 10 to 30 Ni at. percent. In contrast, raising the Ni concentration over this range resulted in a degradation of the glass-forming capacity and the stability of the amorphous phase that was produced; in addition, the crystallization temperature was lower in this composition when compared to the other. This might be due to the formation of a low volume fraction of ultrafine nanoparticles (less than 1 nm) of a metastable phase, which could not be observed by HRTEM because of the low volume fraction.

### 3.3. Cold Spray Coating

#### 3.3.1. Crystal Structure

**Figure 12:**
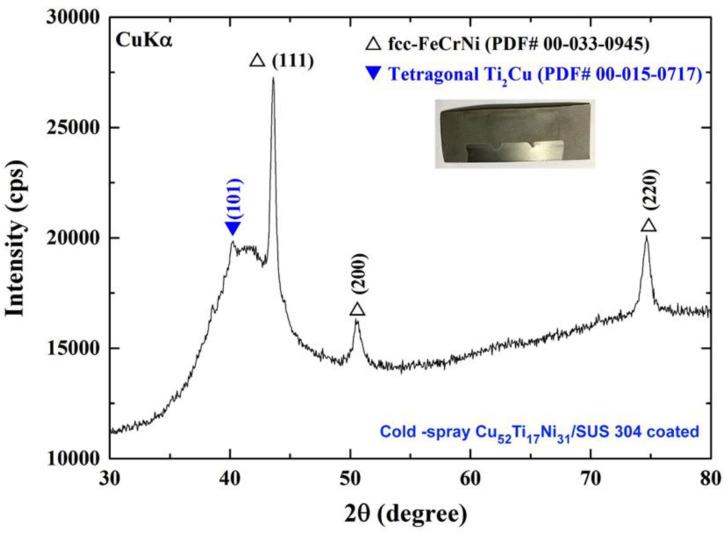
XRD pattern of cold spray metallic glassy coated Cu_52_Ti_17_Ni_31_ powders/SUS304 sheet.

This work utilized the as-synthesized metallic glassy powders obtained after 100 h of MA time as antibacterial feedstock materials for coating stainless steel sheets (SUS304) employing a cold spraying approach carried out in a laboratory setting. According to the objectives of the current study, the cold spraying operation was carried out at a temperature of 600 °C (slightly above the glass transition temperature of the glassy powders). Figure 12 depicts the XRD pattern of Cu_52_Ti_17_Ni_31_ coating that was applied as-cold sprayed. After spraying, the coating deposit displayed a wide halo-diffuse pattern, showing that the cold spraying approach was effective in retaining the amorphous structure of the glassy powders throughout the spraying process.

However, a minor crystalline Bragg peak associated with the crystalline Ti_2_Cu phase was seen with the initial halo (39°), indicating that only a tiny volume fraction of the tetragonal Ti_2_Cu nanocrystalline phase had formed during partial crystallization. The creation of this phase was most likely facilitated by the relatively lengthy amount of time (40 min) required to cool the as-coated system prior to handling. Because the coating used in this work had a thickness of about 1.4 µm, the Bragg peaks of the substrate materials (austenitic SUS304) were diffracted and strongly displayed in the XRD pattern shown in Figure 12.

#### 3.3.2. Morphology and Elemental Distribution

**Figure 13:**
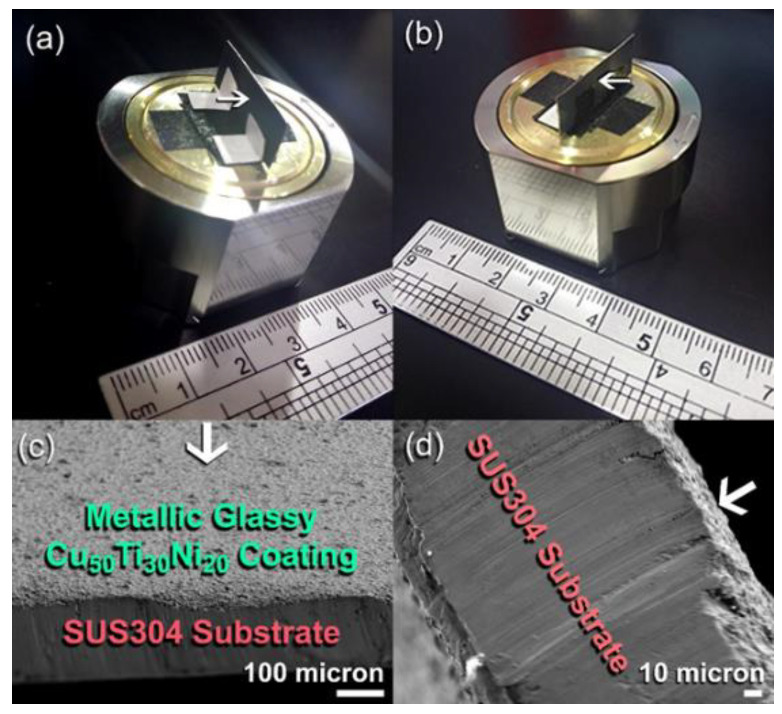
Photos of two-face coated sample of SUS304 by cold spray metallic glassy Cu_52_Ti_17_Ni_31_ powders. The sample was mounted on an SEM-copper sample holder and fixed with carbon tapes (**a**,**b**). The FE-SEM image of the upper and lower parts of the sample are displayed in (**c**,**d**), respectively.

To realize the coating uniformity and distribution of metallic glassy Cu_52_Ti_17_Ni_31_ powder-coated SUS304, a strip of the dual-face coated sample was mounted vertically on an SEM copper sample holder and supported by carbon tape, as illustrated in Figure 13a,b. 

The FE-SEM image of the upper and bottom coated surfaces are shown in Figure 13c,d, respectively. As evidenced by the near thickness values of the coated layer, the coating materials were evenly dispersed over the surface of the substrate, with no uncoated zones visible on the surface of the substrate. 

**Figure 14:**
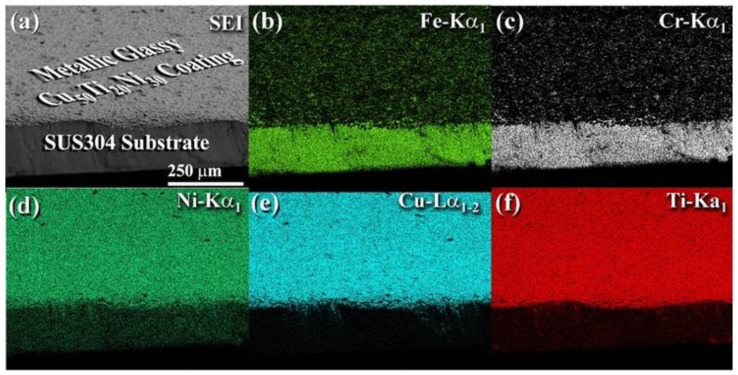
(**a**) Scanning electron image (SEI) and the corresponding EDS elemental mapping of (**b**) Fe, (**c**) Cr, (**d**) Ni, (**e**) Cu, and (**f**) Ti of cold-sprayed metallic glassy Cu_52_Ti_17_Ni_31_ powder-coated SUS304.

Figure 14a–f show the FE-SEM image and the associated EDS mapping for the Fe, Cr, Ni, Cu, and Ti elements of cold spray Cu_52_Ti_17_Ni_31_ coated SUS304, which are displayed together for comparison. In the composite-layer coated material, a substrate made of Fe-18Cr-8Ni (SUS304) was coated with a thin layer of metallic glassy powders (Figure 14a–d) and then coated again with a thin layer of metallic glassy powders (Figure 14d–f).

The EDS findings demonstrated uniform element distributions on the microscopic scale, with no evidence of compositional variations or degradations seen at the macroscale. It is possible to conclude that the cold spraying approach did not result in compositional changes during processing, as demonstrated by the EDS analysis, and that the alloying elements of the metallic glassy phase represented by their three alloying elements were homogeneously distributed without apparent element separation or aggregation. Aside from that, EDS analysis revealed an average atomic Cu/Ti/Ni ratio of 52/17/31, which is extremely close to the nominal average composition of the beginning Cu_50_Ti_20_Ni_30_ powders.

To be clear, the fabrication of thick glassy coating materials with an amorphous random structure is a difficult task that can only be accomplished with metallic glassy systems with a large ΔTx region. This may be attributed to the fact that metallic glassy alloys exhibit superplasticity in the supercooled liquid state as a result of Newtonian viscous flow in the supercooled liquid state. As a result, cold spraying at a temperature that is in a viscous zone when the amorphous Cu_52_Ti_17_Ni_31_ alloy powder alloys exhibit viscous flow resulted in support bonding to the SUS304 substrate when the alloy powder alloys were used.

#### 3.3.3. Nanoindentation

**Figure 15:**
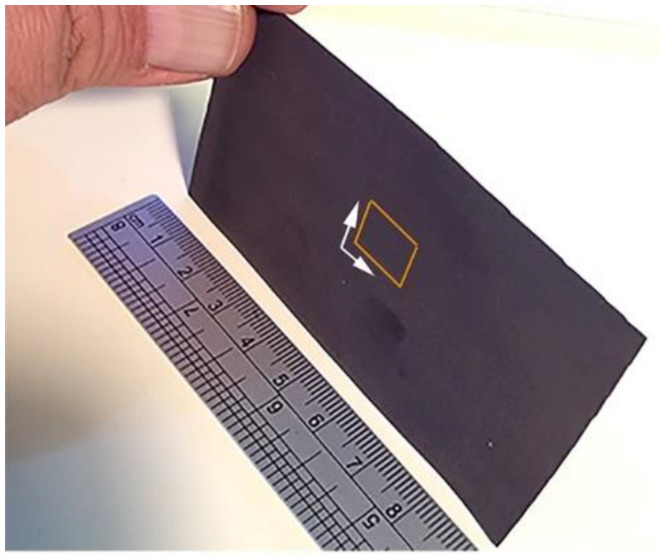
A strip portion (~9 cm × 5 cm) of cold spray metallic glassy Cu_52_Ti_17_Ni_31_ powder-coated SUS304. The small square portion shown in the middle of the strip refers to the sample selected for performing a nanoindentation test, under a load of 400 mN.

**Figure 16:**
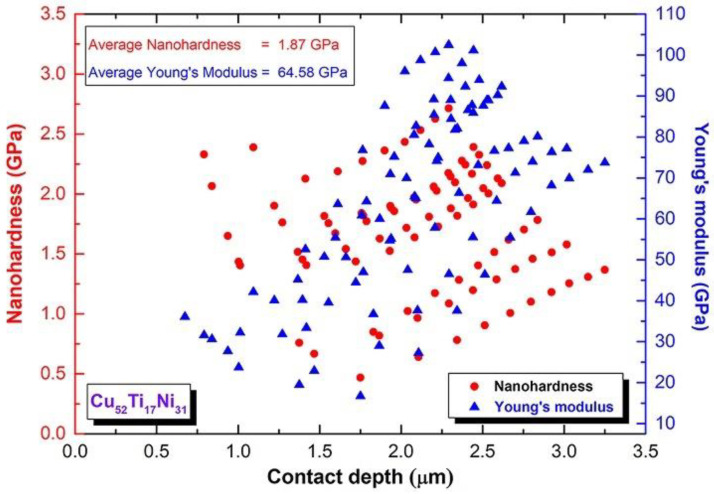
The correlation between nanohardness, contact depth, and Young’s modulus of coated Cu_52_Ti_17_Ni_31_/SUS304.

The nanoindentation test was used to investigate the nanomechanical characteristics of metallic glassy Cu_52_Ti_17_Ni_31_-coated SUS304, which were indexed by nanohardness and Young’s modulus, respectively. Figure 15 shows a small portion of the as-coated SUS304 strip, where the nanoindentation examinations were performed with a load of 400 mN. Figure 16 show the plots of the nanohardness and Young’s modulus acquired after testing a total of 88 points obtained from the tests.

The correlation between nanohardness, contact depth, and Young’s modulus for coated Cu_52_Ti_17_Ni_31_/SUS304 is displayed in Figure 16. The test was achieved at the red-circular symbols shown in Figure 15. As shown in Figure 16, the metallic glassy coating material exhibited very high microhardness values ranging between 2.97 GPa and 3.20 GPa, indicating that it was extremely durable. Furthermore, the value of the Young’s modulus measured at the 88 chosen sites varied from 97 GPa to 111 GPa, depending on the XY coordination of each studied point. The close relationship between nanohardness and Young’s modulus values, as well as the absence of random results, indicate that the cold-sprayed metallic glassy coated SUS304 material has a uniform and homogeneous structure and elemental composition, as indicated by the results of the nanohardness and Young’s modulus measurements.

#### 3.3.4. Pin-on-Disk test

**Figure 17:**
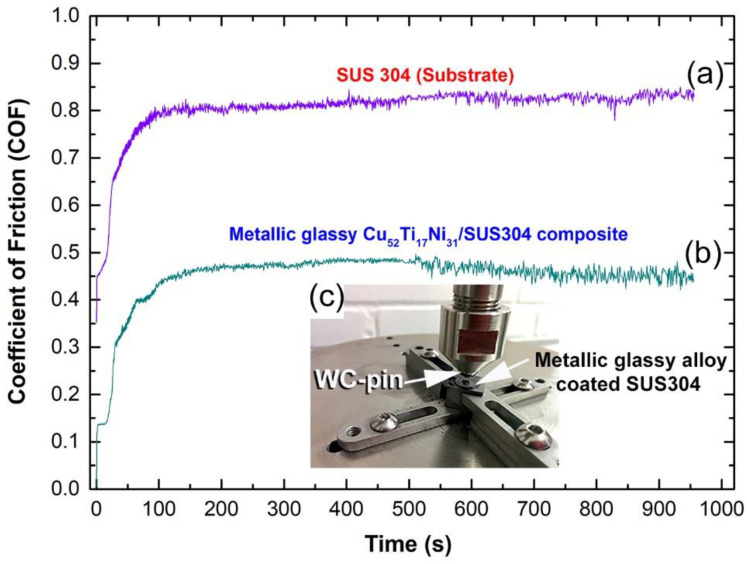
Dynamic COF curves of (**a**) uncoated SUS304 substrate, and (**b**) cold spray metallic glassy Cu_51_Ti_17_Ni_31_ powder-coated SUS304. The wear test was performed with sliding wear pin-on disk testing technique, using a tool-steel ball under a load of 100 N at ambient temperature for 950 s. Powders obtained after ball milling of 100 h. The pin-on-disk experimental setting is shown in (**c**).

The pin-on-disc tribometer was used to evaluate the friction and wear behaviors of a SUS304 substrate before and after cold spray coating with metallic glassy Cu_52_Ti_17_Ni_31_ powders (Figure 17a,b). A WC-Ni composite ball (c) was used to measure the friction and wear behaviors. On the basis of a 100 m total sliding distance, the Figure 17a,b show the change of friction coefficients (COFs) measured for SUS304 and Cu_52_Ti_17_Ni_31_ coating with sliding time at a total sliding distance of 100 m, respectively.

The COFs for SUS304 and the Cu-based coated sample were 0.51 and 0.14, respectively, at the beginning of the sliding time (25 s) of the experiment. According to Figure 17a,b, increasing the sliding duration to 50 s resulted in a rise in the COFs for SUS304 and the metallic glassy coated sample to the levels of 0.70 and 0.33, respectively for SUS304 and the metallic glassy coated sample.

After that, the COFs for SUS304 and Cu_52_Ti_17_Ni_31_ coatings were 0.77 and 0.43, respectively, according to the results. After 150 s of sliding time, the COF for SUS304 reached saturation at 0.83 and remained there without any significant changes until the conclusion of the testing period (Figure 17a). However, as shown in Figure 17b, the coefficient of friction (COF) for the Cu_52_Ti_17_Ni_31_-coated sample decreased to a value of 0.48 over the sliding period ranging from 150 s to 500 s. According to the results, the low COF value recorded for the coating material may be ascribed to its high nanohardness values (3.1 GPa) when compared to the lower hardness (1.29 GPa) obtained for the SUS304 substrate material. According to Figure 17b, the COF measured for the metallic glassy coating material decreased somewhat during the course of the final stage of the sliding period (500 s to 950 s) and toward the conclusion of the test, exhibiting a modest monotonic decrease that reached around 0.45 after 950 s. Several factors contribute to this, including the dense random packed structure, which is known for amorphous structured metals, the consistency of the chemical composition of the powders, and the high hardness value observed in the current investigation for the coating material. When seen in Figure 17b, the presence of such qualities results in a steady and low friction coefficient as the sliding duration is increased.

It has been pointed out by Greer et al. [31] that metallic glasses are more wear-resistant coating candidates and are harder than crystalline counterparts, which makes them a more attractive option. The lack of crystalline long-range periodicity in metallic glasses such as typical dislocation-based deformation routes are avoided. Accordingly, these metastable noncrystalline alloys have higher hardness, wear resistance, and elastic strain limit when compared with traditional crystalline alloys [32].

### 3.4. Microbiological Testing

**Figure 18:**
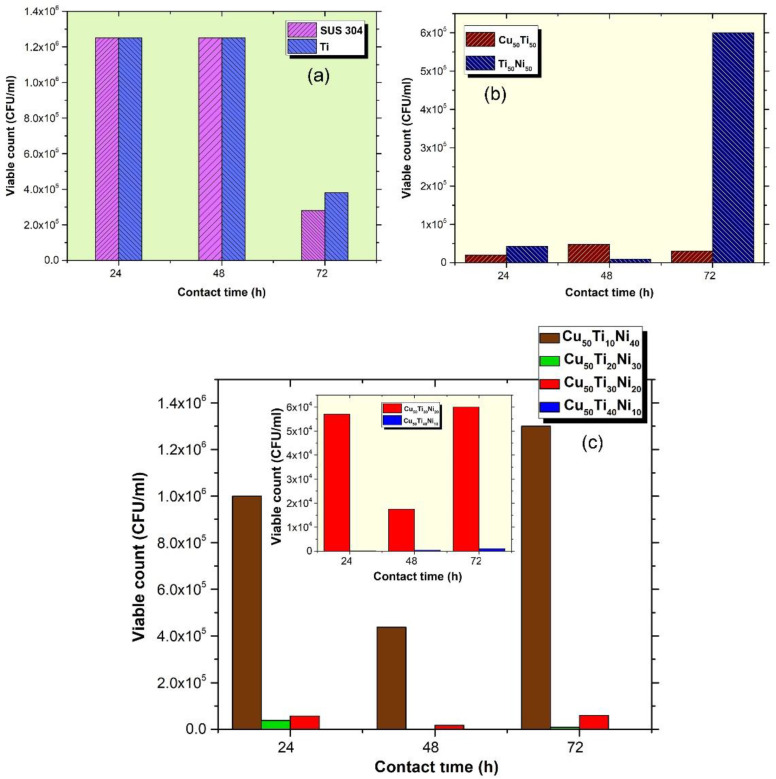
Effect of nanocoating on biofilm formation by *E. coli* (ATCC 25922) after 24 h, 48 h and 72 h (**a**) control coupons, (**b**) binary coated coupons and (**c**) tertiary coated coupons.

The coated and non-coated stainless steel coupons’ adherent *E. coli* counts were determined by vortexing, as mentioned earlier, from the coupons and subsequent viable colony counting. Mean colony counts in both types of coupons are shown in Figure 18. While Figure 18a shows the inhibitory effect of control SUS304 and Ti for comparison purposes, Figure 18b,c show the inhibitory effect of a binary and ternary nanocoating substrate against *E. coli* biofilm formation incubated for 24 h, 48 h and 72 h, respectively. In Figure 18a, there were no statistically significant differences in viable count among the SUS304 and Ti coated coupons (*p* > 0.05). Similarly, there were no statistically significant differences (*p* > 0.05) when comparing the two binary systems including Cu_50_Ti_50_ and Ti_50_Ni_50_ except after 72 h which showed an increase in the viable counts of *E. coli* biofilm cells on Ti_50_Ni_50_-coated coupons. In addition, the binary systems demonstrated a significant inhibitory effect against biofilm formation by *E. coli* (Figure 18b) when compared to SUS304 non-coated coupons and Ti-coated coupons (Figure 18a).

Ternary systems (Cu_50_Ti_30_Ni_20_ and Cu_50_Ti_40_Ni_10_) showed a remarkable statistically significant (*p* < 0.05) inhibitory effect against biofilm formation by *E. coli* especially in the case of Cu_50_Ti_40_Ni_10_ which showed no biofilm formation after 24 h of exposure and less than 103 CFU/mL viable count after 48 and 72 h when compared to control and binary system-coated coupons. On the other hand, Cu_50_Ti_10_Ni_40_ showed no significant inhibitory effect when compared to the SUS304 control coupons.

The significant bactericidal effect of binary systems (CuTi) and ternary systems (CuTiNi) alloy has been shown by the qualitative results obtained against *E. coli* using viable counts (Figure 18). The results presented confirm that Cu_50_Ti_40_Ni_10_ could inhibit bacterial cell adhesion even after 72 h of exposure. In addition, our results demonstrated the significant bactericidal effect against *E. coli* using CuTi and NiTi binary systems.

Maturation of biofilm is a complex process that includes the adhesion of microorganisms including bacteria, viruses and fungi, extracellular polymeric substance (EPS) production followed by the spreading of microorganisms in the environment [33]. In addition, it well known that after the formation of mature biofilm, it is very difficult to eradicate [33,34]. Thus, it is agreed that establishing inhibition techniques to prevent microorganism adhesion as a crucial step. For that reason, research into evaluating existing and novel antibiofilm surface materials for use in clinical and food environments is trending [35].

Kumar et al. reported that noble metals, such as silver, are very effective antimicrobial agents and can be synthesized in situ in a chitosan matrix due to the metal ion chelation capability of chitosan [36]. Moreover, Pontin et al., evaluated the antimicrobial activity of copper surfaces in preventing biofilm formation by Salmonella Enteritidis and to determine their corrosive capacity in the food environment and reported the effectiveness of copper surfaces in reducing *S. Enteritidis* [37]. Mei et al., demonstrated the effective inhibitory effect of titania nanotubes (TiO_2_-NTs) containing Ag against Porphyromonas gingivalis (ATCC 33277) and Actinobacillus actinomycetemcomitans (ATCC 29523) [38]. Furthermore, various metallic glass materials have been developed including 304-Cu, 420-Cu and 317L-Cu antibacterial stainless steels in addition to Ti-6Al-4V-xCu (x = 1, 3, 5) alloy by the addition of Cu to the preparations, and because of the continuous release of Cu ions into the environment, the mentioned materials show very effective antimicrobial properties [38].

Liu et al., developed a Ti-Cu alloy which showed an effective bactericidal effect against the oral bacterial species of *S. mutans* and *P. gingivalis* [7]; Burghardtt et al., demonstrated the significant inhibitory effect of CuTi against *S. aureus* after 24 h [39]. However, in order to produce a novel nanocoated surface with increased antibiofilm properties which would inhibit the adhesion of biofilm formation bacterial cells in clinical and food environments, in the present study, we tried to fabricate a Cu-based metallic alloy including Ti and Ni.

The antibacterial action of Cu-based surfaces and the effect of releasing of Cu^2+^ has been widely studied [7,33,34,35,36,37,38,39,40,41]. The mechanisms of how the Cu-based surfaces affect the bacterial cells are mainly because of the release of copper ions (Cu^2+^) from the metallic surfaces, but also in order to be effective, there should be a direct contact between bacterial cells and surfaces [39]. In addition, many mechanisms have been reported including bacterial injuries and loss of activity when in contact with Cu, damaging of the cell envelope (outer and inner membrane), oxidative damage caused by the production of reactive oxygen species (ROS), enzymatic inhibition and degradation of nucleic acids [41]. It has been reported that the direct contact of Ti-Cu alloy with bacterial cells may led to an enhanced permeability of the cell membrane allowing entry of Cu ions into the cell followed by intracellular material outflow [38].

Although the CuTiNi alloys proved to be exceptional antibacterial inhibitors, especially the Cu_50_Ti_40_Ni_10_ alloy, they need more study to be considered safe for clinical use. The release of a high level of Cu ions may become toxic for human use. On the other hand, Noyce et al. [41], tested Cu-based alloy coatings (61 to 95% Cu) for their ability to reduce the viability of *E. coli* and demonstrated that only >85% Cu content could inhibit biofilm formation on coated stainless steel. In comparison, we indicated a total inhibition with only 50% Cu-based alloy. The limitations of this study include the fact that biofilm formation was only performed in vitro with a monospecies biofilm of *E. coli*. *E. coli* is a commonly used model organism for bacterial adhesion/biofilm accumulation in food environment, yet multispecies need to be tested to reflect bacterial contamination in both clinical and food environments.

## 4. Conclusions

Following low-energy ball milling the elemental metal powders for 100 h, a metastable phase of metallic glassy Cu_50_(Ti_50−x_Ni_x_) was achieved, where x; 10, 20, 30, and 40 alloy powders were obtained. The metallic glassy alloys that were formed had an average particle size of 1.7 µm with spherical-like shape. When heated in a DSC, amorphous Cu_51_Ti_17_Ni_31_ alloy transformed into a liquid glassy phase with a glass transition temperature of 562 °C, with Tx of 641 °C. The homogeneous synthetic metallic glass alloy powders obtained following 100 h of ball milling time were used to treat both sides of SUS304 sheet metal with cold spray at 400 °C. This advanced coating technique did not lead to the crystallization of the formed metastable phase. Metallic glass Cu_51_Ti_17_Ni_31_ coated/SUS304 revealed a high nanohardness value of 3.1 GPa. Additionally, it showed a low value of the coefficient of friction (0.45).

## Data Availability

Not applicable.
